# Challenges in the standardization of in vitro cytotoxicity assays for comparative risk assessment of cold atmospheric pressure plasma devices

**DOI:** 10.1038/s41598-026-45037-8

**Published:** 2026-03-25

**Authors:** Lars Boeckmann, Philipp-Kjell Ficht, Thoralf Bernhardt, Thomas Borchardt, Agnieszka Ossowska, Wieland Milz, Andreas Helmke, Sander Bekeschus, Steffen Emmert

**Affiliations:** 1 Clinic and Policlinic for Dermatology, Venereology, and Allergology, University Medical Center Rostock, Rostock, Germany; 2 Faculty of Engineering and Health, HAWK University of Applied Sciences and Arts Hildesheim/Holzminden/Goettingen, Goettingen, Germany; 3 ZIK Plasmatis, Leibniz Institute for Plasma Science and Technology (INP), Greifswald, Germany

**Keywords:** Cold gas plasma, Standards, Plasma medicine, Cytotoxicity, Treatment parameters, Biological techniques, Biotechnology, Engineering, Medical research

## Abstract

Cold atmospheric pressure plasma (CAP) has emerged as a promising therapeutic modality in wound healing, with multiple devices now certified for clinical use. However, the constructive and functional diversity of CAP technologies poses significant challenges for cross-device comparison in preclinical in vitro studies. In this study, we evaluated how device-specific parameters and experimental conditions influence cytotoxic outcomes across different CAP technologies. Using L929, GM00637, and HaCaT cells, we compared direct treatment with a plasma jet (kINPen^®^ MED) and a surface micro-discharge device (plasma care^®^), revealing significant differences in the reduction of metabolic activity under otherwise identical conditions. Assessment of treatment geometry—specifically the radius of circular motion of the plasma jet—significantly affects metabolic activity, even at identical exposure times. To standardize conditions across devices, we further investigated an indirect treatment approach using a metal grid to generate plasma-conditioned PBS. However, we found a non-linear relationship between liquid volume, treatment time, and biological outcome. Moreover, indirect treatment excludes short-lived reactive species and non-chemical plasma components, limiting its biological relevance. Our findings demonstrate that neither direct nor indirect treatment protocols reliably enable cross-device comparisons in vitro. We therefore advocate for transparent, comprehensive reporting of all device and experimental variables, rather than pursuing a single standardized protocol. This enables meaningful data integration and cross-study comparisons, even when protocols differ.

## Introduction

Cold atmospheric pressure plasma (CAP) has emerged as a promising modality in plasma medicine, transitioning from fundamental research to clinical applications^1,2^. Over the past decade, clinical evidence supporting CAP’s efficacy in wound healing has grown substantially, with at least six CAP devices now CE-certified for medical use in Europe and numerous others under investigation in preclinical and translational studies^3–9^. These devices span a range of technologies, including plasma jets, plasma torches, surface dielectric barrier discharges (SDBD), and volume dielectric barrier discharges (VDBD), each differing in geometry, operating principles, and treatment dynamics^10,11^.

Plasma jet devices generate CAP internally via noble gas (e.g., argon, helium) flow, delivering the CAP to the target surface through a continuous gas stream^12^. These systems typically feature small active treatment areas. Plasma torch devices can produce thermal or non-thermal discharges at controlled distances from the target using spacers to maintain safe tissue temperatures, though they still rely on gas flow^13^. SDBD and VDBD devices operate without external gas flow, generating plasma in ambient air between a dielectric barrier and the target (in VDBD) or in a confined chamber (in SDBD)^1,14^. Devices based on these technologies often have larger treatment areas and hence are well-suited e.g. for the treatment of large chronic wounds.

The diversity of CAP technologies and devices in clinical studies and especially in pre-clinical in vitro experiments brings about significant challenges in comparing results across studies^15–18^. Variability in treatment geometry, gas flow, liquid displacement, and the absence of standardized cell culture platforms hinder cross-device validation. While the German DIN SPEC 91315:2025-07 provides comprehensive guidelines for characterizing CAP sources for medical use—including physical parameters (UV emission, gas composition, electrical current) and biological effects (reactive species detection, cytotoxicity)—practical challenges of in vitro experimentation persist^19^.

A key limitation lies in the direct application of CAP to cell cultures: plasma jets induce mechanical stress from gas flow, while SDBD/VDBD devices require specific electrode configurations or chamber setups. Moreover, the mismatch between device-specific treatment areas and standard cell culture vessels complicates direct comparisons. To address these issues, indirect treatment strategies—where CAP is applied to a liquid medium (e.g., PBS) via a metal grid, generating “conditioned PBS” that is then used to treat cells—may provide a standardized procedure independent of physical variables such as gas flow and size of the active treatment area.

However, the influence of critical parameters such as liquid volume, treatment duration, and treatment area on the biological potency of conditioned PBS remains poorly understood. In this study, we systematically evaluated how direct and indirect CAP treatment settings affect in vitro outcomes across different cell types and plasma technologies. We specifically tested whether an indirect treatment approach using a metal grid and standardized liquid can enable reliable comparisons of CAP effects from different devices. Thereby, this study provides novel insights into the standardization of plasma medicine research to facilitate the comparison of results from in vitro experiments with different CAP technologies.

## Results

### Differential cytotoxic effects of direct CAP treatment across device types

**Fig. 1:**
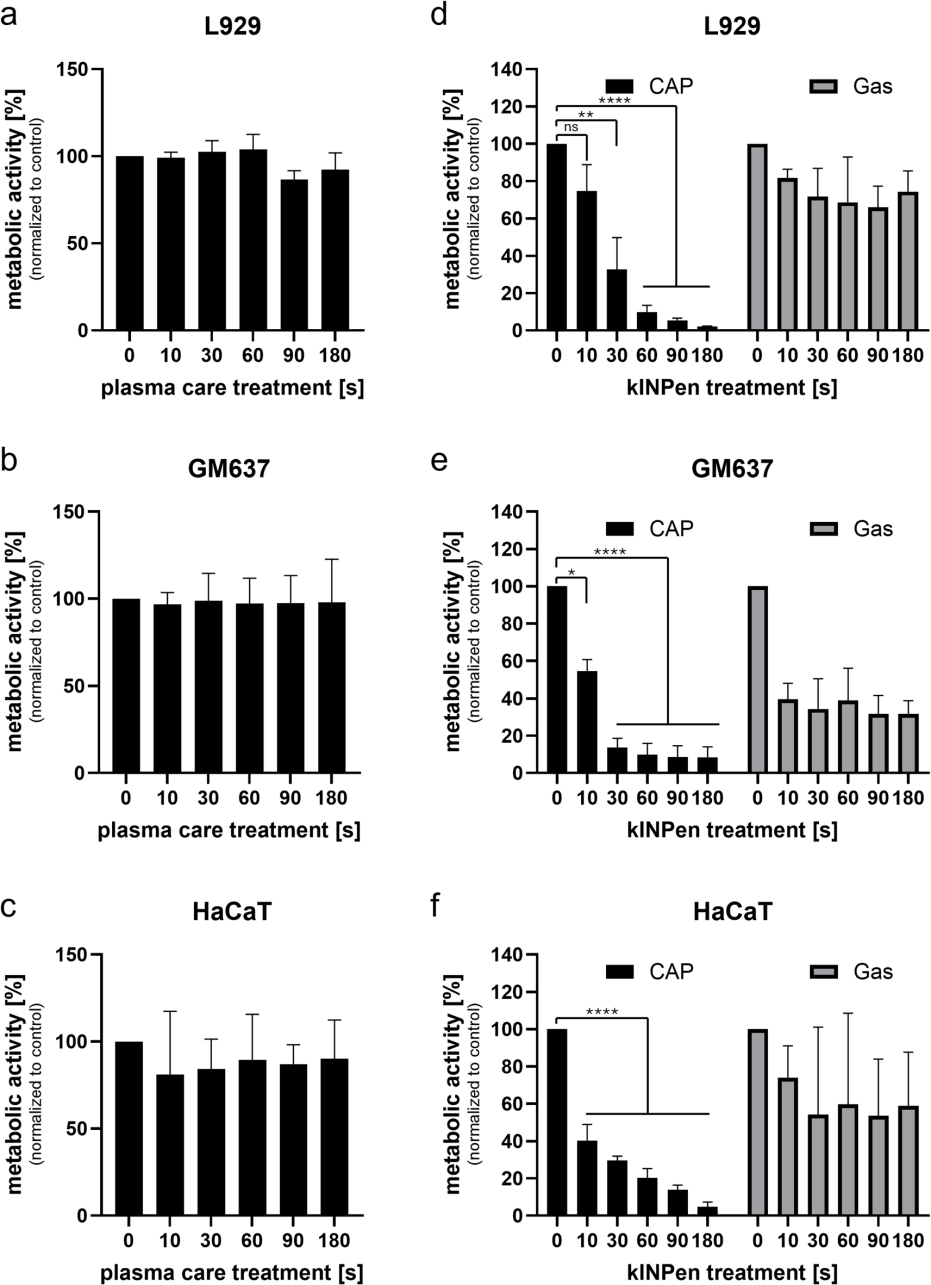
Metabolic activity of L929, GM00637, and HaCaT cells after cold atmospheric pressure plasma (CAP) or argon gas exposure (0–180 s). (**a**–**c**) Cells were treated with plasma care^®^ device. (**d**–**f**) Cells were treated with kINPen MED^®^ device. Data are normalized to untreated controls and represent means ± SD from independent experiments (plasma care: *n* = 2; kINPen: *n* = 4 for CAP and *n* = 2 for Gas). Statistical significance was tested for CAP treatment with kINPen: ns = not significant, *p < 0.05, **p < 0.01, ***p < 0.001, ****p < 0.0001 (one-way ANOVA with Dunnett’s post-hoc test).

To establish a standardized in vitro experimental framework for comparative evaluation of different cold atmospheric pressure plasma (CAP) technologies, we exposed three distinct cell lines—L929 (murine fibroblasts), GM00637 (human fibroblasts from a healthy donor), and HaCaT (non-malignant immortalized human keratinocytes)—to CAP generated by two clinically relevant devices: a surface micro-discharge device (plasma care) and a plasma jet (kINPen MED). All treatments were performed in 35-mm cell culture dishes under consistent conditions. For the plasma jet, we included an argon gas-only control (no plasma discharge) to isolate the effects of gas flow from those of the generated plasma (Fig. 1).

No substantial reduction in metabolic activity was observed in any of the tested cell lines after treatment with the plasma care device, regardless of exposure time. In contrast, the kINPen MED induced a time-dependent decrease in metabolic activity in all three cell lines, with significant effects detectable after just 10 s of treatment for GM00637 and HaCaT and after 30 s for treatment for L929. Notably, a clear reduction in metabolic activity was also observed in cells exposed to argon gas alone—though the effect was consistently less pronounced than with active CAP. These findings underscore the importance of including a gas-only control when using plasma jet devices, as gas flow alone can induce measurable biological effects.

### Treatment geometry significantly influences in vitro outcomes with plasma jet devices

**Fig. 2:**
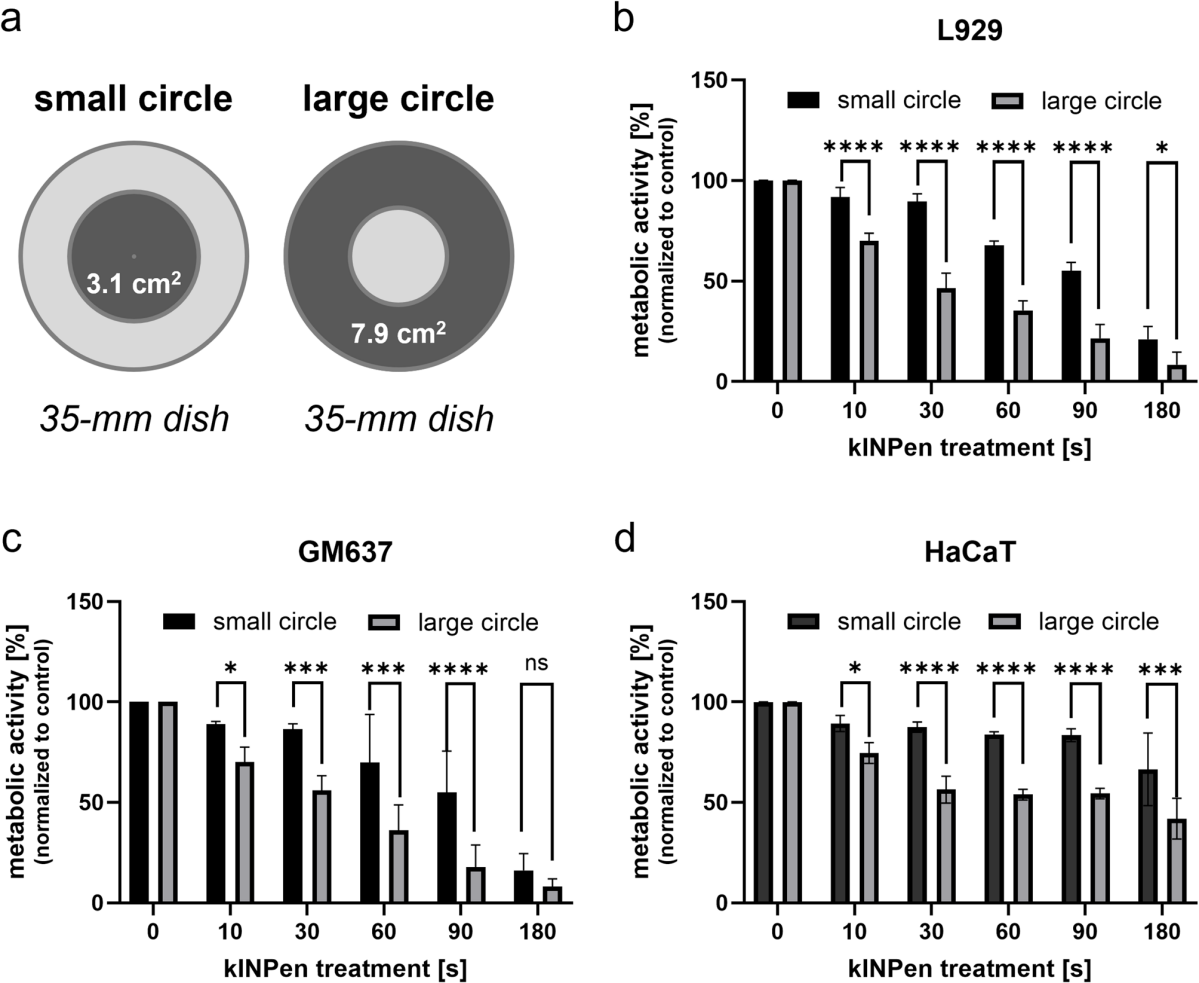
Comparison of circular treatment patterns for kINPen MED plasma jet on 35-mm dishes. (**a**) Schematic of treatment geometries. (**b**–**d**) Metabolic activity of L929, GM00637, and HaCaT cells after treatment with varying circle radii. Data are normalized to untreated controls and represent means ± SD from three independent experiments (*n* = 3). Statistical significance: ns = not significant, *p < 0.05, **p < 0.01, ***p < 0.001, ****p < 0.0001 (Ordinary two-way ANOVA with Šídák’s post-hoc test).

The active treatment area of the kINPen MED plasma jet is approximately 10 mm in diameter. During treatment, the handpiece was moved in a circular path over the 35-mm dish. To assess the influence of treatment geometry, we compared two circular motion settings: a small circle with a radius of 5 mm around the center of the dish (treated area: ~3.1 cm²), and a large circle with a radius of 12.5 mm around the center of the dish (treated area: ~7.9 cm²) (Fig. 2a).

Both settings resulted in a time-dependent decrease in metabolic activity across all cell lines (Fig. 2b–d). However, the rate of metabolic decline was significantly faster in the large-circle setting, indicating that the extent of direct plasma exposure—determined by the radius of the circular motion—has a substantial impact on treatment efficacy. This highlights a critical experimental variable: the treatment geometry of plasma jets can significantly alter biological outcomes, even when treatment time and device settings are identical.

### Non-linear scaling of cytotoxicity with liquid volume and treatment time compromises indirect treatment for device comparisons

Given the challenges associated with direct plasma treatment—such as liquid displacement, mechanical stress from gas flow, and the lack of standardized cell culture vessels matching different applicator sizes—we explored an indirect treatment approach using a metal grid placed over a PBS-filled dish. The grid serves as a counter electrode, allows plasma penetration into the liquid, and enables standardized treatment of a defined liquid volume. The resulting plasma-treated PBS (conditioned PBS) was then used to treat cell cultures.

**Fig. 3:**
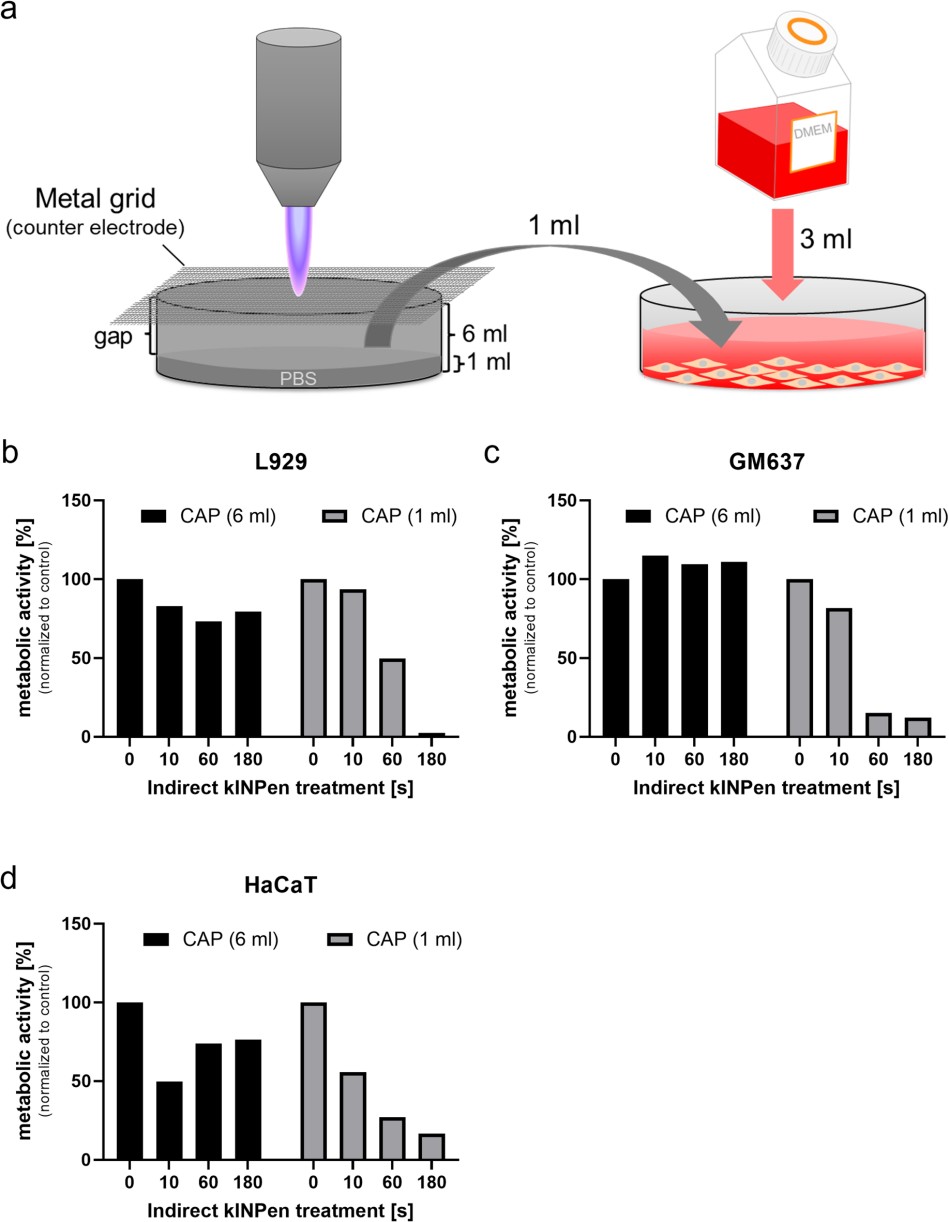
Indirect CAP treatment through metal grid. (**a**) Schematic of setup. (**b**–**d**) Metabolic activity of cell lines after treatment with 1 ml CAP-conditioned PBS from 1 ml or 6 ml PBS volumes. Data normalized to untreated controls (*n* = 1 for each of three independent cell lines).

We first tested two PBS volumes in 35-mm dishes: 1 ml (small volume, large gap of about 7 mm between grid and liquid) and 6 ml (large volume, small gap of about 2 mm). When 1 ml of CAP-treated PBS was applied to cells, a time-dependent reduction in metabolic activity was observed in all three cell lines (Fig. 3b–d). In contrast, 1 ml of PBS from the 6 ml treatment showed no clear cytotoxic effect. This suggests that even if there is a gap between the metal grid and the liquid, the treatment of a small volume of PBS with a plasma jet device through a metal grid is sufficient to generate conditioned PBS with cytotoxic properties on cell cultures. However, taking only one sixths of a larger liquid volume, although treated with a minimal gap, was not sufficient to induce relevant cytotoxic effects.

**Fig. 4:**
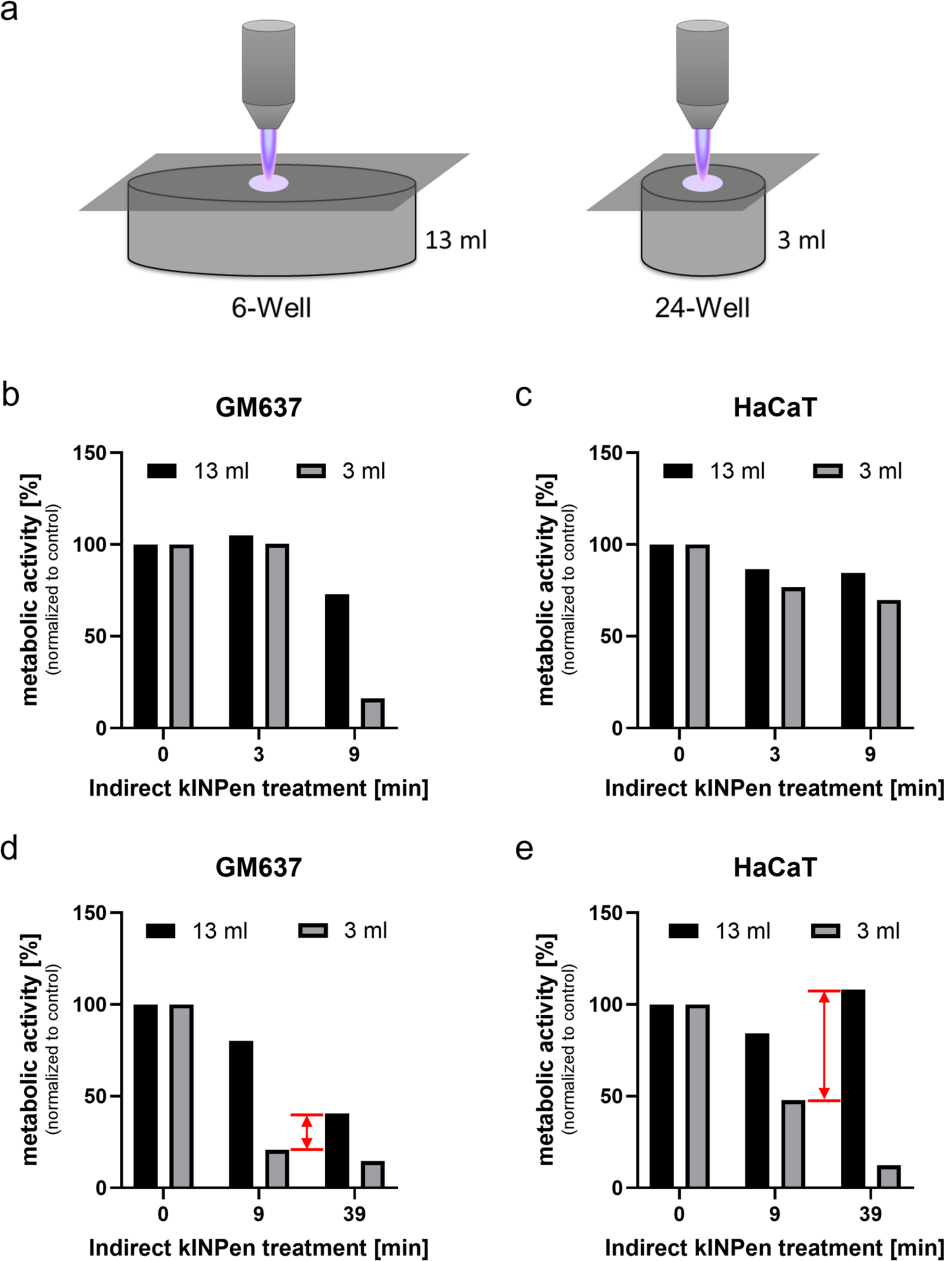
Volume-dependent efficacy of indirect CAP treatment. (**a**) Schematic of 6-well vs. 24-well setup. (**b**–**e**) Metabolic activity after treatment with 1 ml of CAP-conditioned PBS from 6-well and 24-well plates. Data normalized to untreated controls (*n* = 1 for each of two independent cell lines).

To further investigate the relationship between treated volume and efficacy, we compared CAP treatment of PBS in 6-well (13 ml) and 24-well (3 ml) plates, ensuring identical treatment area, distance, and no significant gap between grid and liquid (Fig. 4a). After 9 min of CAP exposure, a slight reduction in metabolic activity was observed in fibroblasts (GM00637) and keratinocytes (HaCaT) in the 6-well plate, while a clear decrease was only seen in fibroblasts in the 24-well plate (Fig. 4b–c).

Given that the 6-well volume is 4.33 times larger than the 24-well volume, we hypothesized that a 4.33-fold increase in treatment time (9 min × 4.33 ≈ 39 min) would yield comparable cytotoxicity. However, after 39 min of treatment, the conditioned PBS from the 6-well plate still induced markedly weaker effects than the conditioned PBS from the 24-well plate (Fig. 4d–e, red arrows). This indicates that the relationship between treatment volume and biological effect is not linear, and larger volumes require disproportionately longer treatment times to achieve equivalent effects.

Collectively, these results demonstrate that multiple factors—gas-only effects, treatment geometry, and liquid volume—significantly influence in vitro outcomes. Despite the promise of indirect treatment approaches, no simple scaling rule based on treatment area, volume, and time can reliably predict relative cytotoxicity across different plasma devices or geometries. Therefore, direct comparisons of CAP technologies in vitro remain highly challenging and must be interpreted with caution.

## Discussion

This study demonstrates that even minor variations in CAP device design and treatment geometry can lead to substantial differences in cytotoxic outcomes, highlighting a critical barrier to standardization and cross-device comparison in plasma medicine. Furthermore, our findings reveal that a simplified indirect in vitro treatment setup—while intended to standardize conditions—is insufficient to reliably assess or compare relative cytotoxicity across different plasma technologies.

To date, only a limited number of studies have directly compared in vitro results from cell culture experiments using multiple CAP devices^20–24^. Bakhtiyari‑Ramezani et al. compared direct and indirect CAP effects of two plasma jet devices with different internal electrode configurations^22^. One was operated with helium and one with argon gas to treat human glioblastoma cells. Both devices were used with 2 slm gas flow and had a similar active treatment area, allowing for treatment of cell cultures in 96-well plates. Similarly, another study evaluated two argon- (2.6 slm and 4.0 slm) and one helium-fed plasma jets (2.6 slm) by treating adherent cells in suspension, thereby minimizing mechanical stress and enabling better matching of cell culture vessels^24^. These approaches were feasible due to the similarity in device type and treatment parameters. However, such comparisons become significantly more challenging when extending to devices with fundamentally different operating principles—such as a plasma jet versus a surface dielectric barrier discharge (SDBD)—where differences in active treatment area, plasma generation mechanism, and gas dynamics are pronounced.

Arndt et al. addressed this challenge by comparing a plasma jet and a plasma torch device using a meta-analytical approach based on published molecular and cellular data^23^. While this strategy offers valuable insights into general mechanisms, it precludes direct quantitative comparisons of cytotoxicity due to inconsistent experimental setups across studies. An in vitro study by Bekeschus et al. comparing the cytotoxic efficacy of a floating-electrode DBD and a plasma jet was performed across two laboratories and demonstrated a way to compare devices by determining “50% inhibitory concentrations” based on plasma treatment energy (DBD) or treatment time (jet)^21^. However, this study also revealed that the liquid volume covering the cells has a significant effect on the treatment outcome. While small volumes of liquid completely inhibited the cytotoxic efficacy of the DBD treatment, an adequate layer of liquid was essential for the treatment with the plasma jet to protect cells from drying effects of the gas stream.

A standardized protocol for comparing antimicrobial properties of CAP from different devices was proposed by Mann and colleagues^25^. In this study, a plasma jet and a surface dielectric barrier discharge (SDBD) device were compared. In contrast to in vitro studies with adherent cell cultures the bacteria and yeast strains were treated on solid agar plates, enabling quantification via colony-forming units per square centimeter. This approach avoids the confounding effects of liquid-mediated species diffusion, which can obscure spatially resolved effects in cell culture. Another study investigated the anti-microbial efficacy of a SDBD plasma device and two surgical plasma tools (J-Plasma, Piezo Brush PZ3) on 5 mm × 5 mm biofilms^26^. While the small size of the biofilms allowed full treatment coverage with each device, it raises questions about scalability. While the same treatment time would have been sufficient for the SDBD to treat a larger biofilm, extended treatment times would be required for other devices with smaller active treatment areas, and, hence, the relative outcome might have been different. This underscores the fundamental challenge of identifying a scalable standardized in vitro setting for the comparison of a multitude of devices and technologies.

To circumvent the limitations of direct treatment—such as gas-induced mechanical stress and liquid displacement—we evaluated an indirect approach using a metal grid to generate conditioned PBS. However, our results revealed a non-linear relationship between treated volume, treatment time, and biological outcome. For instance, treating 13 ml of PBS required disproportionately longer exposure times than 3 ml and did not achieve comparable cytotoxicity, suggesting that scaling by volume and time alone is inadequate. In particular the long treatment times required to treat relatively large volumes of liquid render this approach not practical. The non-linear behavior likely arises from changes in plasma-liquid interaction dynamics, including electric field distribution, reactive species diffusion, and heat dissipation as liquid volume increases. Moreover, indirect treatment inherently excludes short-lived reactive species and non-chemical components such as electric fields and electromagnetic radiation, which may contribute significantly to direct biological effects. Differences between direct and indirect treatment with reduced efficacy of indirect treatment have been reported before^22,27^.

While all experiments and analyses have been conducted with great care, this study comes with some limitations. Some experiments—as indicated in the figure legends—have not been performed in three independent replicates. However, we believe that conclusions drawn from the results are justified even if statistical analysis were not always possible, as similar results with clear effects have been observed for different cell lines.

Together, these findings underscore the profound complexity of standardizing in vitro experiments in plasma medicine. While indirect treatment strategies—such as generating conditioned PBS via plasma exposure through a metal grid—offer a promising avenue for minimizing device-specific variables such as gas flow and treatment area mismatch, our results indicate that these approaches do not inherently yield comparable outcomes across experimental conditions.

## Conclusion

Our findings underscore that the biological effects of CAP are susceptible to both device-specific design and experimental execution, challenging comparative in vitro studies. Rather than pursuing a single standardized protocol, which may be impractical across diverse plasma technologies, we advocate for rigorous, transparent reporting of all relevant experimental parameters.

To enable meaningful cross-study comparisons and reproducibility, we propose that all publications in plasma medicine should report a minimal, standardized set of data, including device specifications (technology type, voltage, frequency, gas type and flow rate, active treatment area), treatment geometry (distance, motion pattern, treated area), and experimental details (liquid volume and composition, cell type and density, treatment time and frequency, post-treatment incubation period). This approach does not aim to replace experimental diversity but to improve comparability, which allows researchers, reviewers, and clinicians to assess the context and reproducibility of results, even when protocols differ. By embracing transparency cumulative data will provide an evidence-based foundation for clinical translation. Crucially, even with robust preclinical data and transparent reporting, the clinical use of any new CAP device must be preceded by rigorous, well-designed clinical trials to ensure safety, efficacy, and reproducibility in human patients.

## Materials and methods

### Cell lines and cell culture

All cell lines (L929 fibroblasts [Cell Lines Service: #400260, CVCL_0462], GM00637 fibroblasts [Coriell Institute for Medical Research, CVCL_7297], HaCaT keratinocytes [Cell Lines Service: #300493, CVCL_0038]) were selected according to the DIN SPEC 91315:2025-07^19^ and cultured in Dulbecco’s Modified Eagles Medium (DMEM, with L-Glutamin, 4.5 g/l Glucose) supplemented with 10% fetal bovine serum (FBS) and penicillin/streptomycin (100 units/ml penicillin, 100 µg/ml streptomycin; Merck KGaA, Darmstadt, Germany) at 37 °C in humidified air with 5% (v/v) CO_2_. All cell lines were tested for mycoplasma contamination using the MycoAlert^®^ Mycoplasma Detection Kit (Lonza, Basel, Switzerland) prior to use.

### Plasma devices

The medical plasma jet device (kINPen^®^ MED, neoplas med GmbH, Greifswald, Germany) and a medical surface dielectric barrier discharge (SDBD) also known as surface micro-discharge (SMD) device (plasma care^®^, terra plasma medical GmbH, Garching, Germany) were used to generate cold atmospheric pressure plasma (CAP).

The kINPen^®^ MED was operated at 1 MHz with a power of 1 W delivered to the argon gas discharge to generate a cold plasma (argon gas: 99.9999% purity; Air Liquide, Germany). The argon gas flow rate was set to 5 standard liters per minute (slm) and the operating distance from the capillary outlet of the applicator to the surface of the target (liquid or metal grid) was approximately 10 mm to create conductive treatment conditions^28^. In addition to untreated samples, samples were exposed to the carrier gas without electrical energy transfer to control for effects of gas flow (gas control).

The plasma care^®^ was operated at ambient air. By applying a voltage of 3.5 kV_pp_ (kilovolts peak-to-peak) at a frequency of 4 kHz micro discharges were produced on the mesh grid of the structured electrode to produce the cold plasma. A spacer keeps the plasma at a distance of 8 mm from the target and provides a confined space for reactive gas species to diffuse to the surface of the target. A special spacer for research purposes was provided by the manufacturer. This spacer was designed to fit the size of a 35-mm cell culture dish and—in contrast to a standard spacer for medical use—it allowed for continuous treatment.

### Direct CAP treatment

For direct treatment 2.5 × 10^5^ cells were seeded in complete DMEM into 35-mm cell culture dishes and incubated overnight at 37 °C and 5% CO_2_. After 24 h attached cells were washed twice with PBS. Then cells were covered with 1 ml PBS and treated with CAP. For the treatment with the plasma jet (kINPen^®^ MED) the applicator was mounted on a CNC-STEP xyz-table and moved in a circular path with a radius of 5 mm (small circle) or 12.5 mm (large circle), centered on the dish. The plasma care^®^ device was placed over a 35-mm dish leaving no gap for the generated reactive species to escape until treatment was finished and the device lifted from the dish.

Immediately after the CAP treatment, 3 ml DMEM high glucose (4.5 g/l Glucose) with L-glutamine supplemented with 1% penicillin/streptomycin and an increased FBS content of 13% to achieve a final concentration of 10% FBS were added to the treated PBS and cells were incubated at 37 °C and 5% CO_2_. After incubation for 48 h the metabolic activity was measured (see below).

### Indirect CAP treatment

For indirect treatment varying volumes of PBS were treated with CAP through a grounded metal grid (Aaronia Shield^®^ ULTRA, Aaronia AG, Strickscheid, Germany) in different well-formats as indicated in the results section. One milliliter of the treated/conditioned PBS was then transferred on cells in a separate 35-mm cell culture dish. The transfer was performed immediately after plasma treatment to minimize reactive species decay. The cells were seeded, cultured, and washed as described above for the direct treatment. After transferring the conditioned PBS onto the cells, 3 ml DMEM high glucose (4.5 g/l Glucose) with L-glutamine supplemented with 1% penicillin/streptomycin and an increased FBS content of 13% to achieve a final concentration of 10% FBS were added and cells were incubated at 37 °C and 5% CO_2_. After incubation for 48 h the metabolic activity was measured (see below).

### Analysis of metabolic activity

The metabolic activity of human cell cultures was determined after treatment using the CellTiter 96^®^ Non-Radioactive Cell Proliferation Assay (MTT, Promega GmbH, Walldorf, Germany) according to the manufacturer’s protocols. After CAP treatment and incubation for 48 h, the medium was replaced with 1 mL fresh medium and 100 µL Dye Solution (MTT) was added. The reaction was stopped after 4 h incubation at 37 °C by adding 1 mL Stop-Solution. The formation of a formazan product was measured 24 h later at a wavelength of 540 nm using a GloMax^®^ Discover plate reader (Promega GmbH, Walldorf, Germany). For this, 200 µl (three technical replicates) were transferred from the 35-mm dish into a 96-well plate.

### Statistical analysis

GraphPad Prism, Version 10.6.1 (GraphPad Software Inc, San Diego, CA, USA) was used to visualize data and to perform statistical analyses. The number of replicates and the statistical test for different experiments are indicated in the figure legends.

### Use of artificial intelligence in manuscript preparation

The preparation and refinement of this manuscript were supported by the use of an artificial intelligence (AI) tool (Academic Cloud, Chat AI, Qwen 3 30B A3B Instruct 2507) to assist in enhancing clarity and coherence. AI was employed to suggest improvements in sentence structure, vocabulary selection, and logical flow. All AI-generated content was critically reviewed, edited, and validated by the authors to ensure scientific accuracy, factual correctness, and alignment with the original data and intent. The use of AI was limited to language refinement and did not involve the generation of scientific hypotheses, data interpretation, or experimental design. The authors take full responsibility for all content, conclusions, and decisions made throughout the research and writing process.

## Data Availability

The datasets generated and analyzed during the current study are available from the corresponding author on reasonable request.
